# A short review: Recent advances in electrospinning for bone tissue regeneration

**DOI:** 10.1177/2041731412443530

**Published:** 2012-04-04

**Authors:** Song-Hee Shin, Odnoo Purevdorj, Oscar Castano, Josep A Planell, Hae-Won Kim

**Affiliations:** 1 Institute of Tissue Regeneration Engineering (ITREN), Dankook University, Cheonan, South Korea; 2 Department of Nanobiomedical Science and WCU Research Center, Dankook University, Cheonan, South Korea; 3 Biomaterials for Regenerative Therapies, Institute for Bioengineering of Catalonia (IBEC), Barcelona, Spain; 4 Centro de Investigación Biomédica en Red en Bioingeniería, Biomateriales y Nanomedicina (CIBER-BBN), Zaragoza, Spain; 5 Technical University of Catalonia (UPC), Barcelona, Spain; 6 Department of Biomaterials Science, School of Dentistry, Dankook University, Cheonan, South Korea

**Keywords:** Electrospinning, hard-tissue regeneration, composites, drug delivery, stem cells

## Abstract

Nanofibrous structures developed by electrospinning technology provide attractive extracellular matrix conditions for the anchorage, migration, and differentiation of tissue cells, including those responsible for the regeneration of hard tissues. Together with the ease of set up and cost-effectiveness, the possibility to produce nanofibers with a wide range of compositions and morphologies is the merit of electrospinning. Significant efforts have exploited the development of bone regenerative nanofibers, which includes tailoring of composite/hybrid compositions that are bone mimicking and the surface functionalization such as mineralization. Moreover, by utilizing bioactive molecules such as adhesive proteins, growth factors, and chemical drugs, in concert with the nanofibrous matrices, it is possible to provide artificial materials with improved cellular responses and therapeutic efficacy. These studies have mainly focused on the regulation of stem cell behaviors for use in regenerative medicine and tissue engineering. While there are some challenges in achieving controllable delivery of bioactive molecules and complex-shaped three-dimensional scaffolds for tissue engineering, the electrospun nanofibrous matrices can still have a beneficial impact in the area of hard-tissue regeneration.

## Introduction

**Figure 1.**
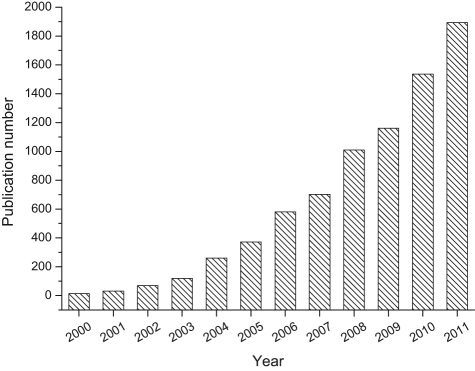
Plot of annual research publications concerning electrospinning studies. Search data from http://www.scopus.com, years 2000–2011, using “electrospinning” or “electrospun,” as article title, abstract, or keywords.

Since the invention of electrospinning in the early 20th century,^1^ there has been enormous activity in this area during the last two decades,^2–5^ with more than 1500 annual reports and 15,000 publications being written on the subject (Figure 1). This technology has also been considered as highly useful for fabricating scaffolds for culture of tissue cells and the treatment of damaged and diseased tissues, including blood vessels, muscles, skins, tendons, ligaments, cartilage, nerves, and bones.^6,7^

The hierarchical structures of electrospun materials, namely, continuous nanostructured fibers with diameters of tens of nanometers over a few micrometers, are considered to mimic the native tissue structures that comprise matrices surrounding tissue cells.^7^ When compared to the flat surfaces of dense materials, nanofibrous networks feature substantially increased surface area with directional fiber alignment, which helps cells to recognize more sites for adherence and guides them to spread and migrate in specific directions.^8^ Technological advances in electrospinning have also sought to improve the capacity of the nanostructured fibrous matrices for tissue-regeneration processes. For example, nanofibers have been aligned to guide neural or muscle cells,^9^ designed with a core–shell structure to incorporate therapeutic drugs inside,^10^ and tailored to have hybrid compositions with appropriate properties such as mechanical integrity for hard tissues.^11^

Among areas relevant to tissue regeneration, this review focuses on systems that target bone tissue. This is the tissue in which extracellular matrices (ECMs) are essentially composed of organic and inorganic nanocomposites. The artificial materials must be designed to have the mechanical properties needed to sustain loads and should be favorable for recruiting cells specific for mineralizing tissues.^12^ Recent advances in electrospinning technology related to the regeneration of calcified hard tissues are summarized. In this review, the design of electrospinning equipment is briefly introduced, followed by a review of newly developed materials for target tissues, potential therapeutic applications, and perspectives regarding future directions.

## Electrospinning designs

### General setup

**Figure 2.**
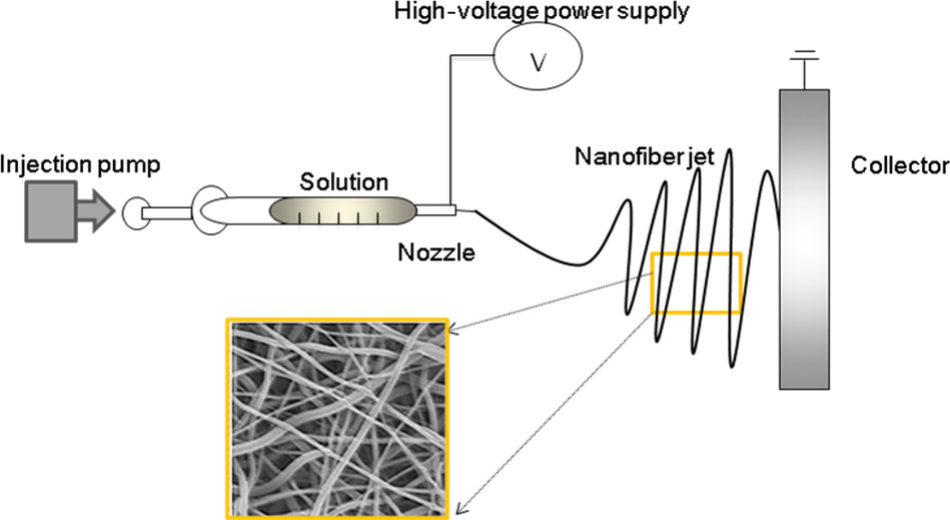
Illustration of electrospinning setup consisting of a power supply, injection pump and nozzle, and conducting collector.

The essential components of electrospinning equipment consist of a power supply, injection pump and nozzle, and a conducting collector, as described in Figure 2. The solution or slurry kept in a syringe is injected through a nozzle by the application of DC electrical power and then collected on a conducting substrate. In an electrical field, the solution at the nozzle tip becomes unstable as a result of surface tension and the electrical potential, and a sudden jet spinning is achieved upon overcoming the surface tension.^3,13^ The nanofibers generated by the electrospinning have diameters ranging from tens of nanometers to several micrometers. The power (voltage and current), injection rate, nozzle capacity, collector design, and environmental factors such as temperature and humidity are possible variables that must be controlled. Above all, the type of solution (materials) and the fluid properties such as viscosity, surface tension, and vapor pressure should be carefully adjusted to form a continuous and homogeneous-sized fiber. A new design of the apparatus is mainly useful to achieve specific forms of nanostructured fibers, such as aligned, core–shell structured, or macroporous nanofibers.

### Alignment of nanofibers

**Figure 3.**
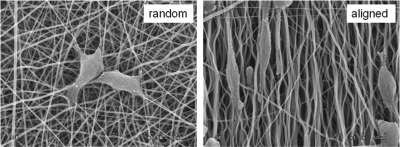
Random and aligned electrospun nanofibers (PLCL synthetic biopolymer) influencing the direction of cellular spreading and elongation. Courtesy of Dr. Park JS in Medical College of Dankook University PLCL: poly(lactide-*co*-caprolactone).

When the electrospun fiber is aligned, it is considered to guide tissue cells in the direction of the fiber, and this is particularly relevant in areas of aligned tissues, including nerves and muscles. In the case of hard tissues, such as bone, the alignment of collagenous fibers is of special importance. Calcified bone tissue exhibits different mechanical properties depending on the collagen alignment in the native structure.^14^ It has been accepted that strength is higher along the direction parallel to the fiber alignment than along the direction perpendicular to fiber alignment.^3^ Moreover, compared to randomly oriented fibers, the aligned fibers exhibited significantly improved resistance to tensile stress (~8–10 fold more) when tested parallel to the fiber alignment.^15^ This alignment of fibers influences cell behavior starting from the initial cell spreading and elongation (as shown in Figure 3), which in turn influences matrix synthesis and possibly differentiation and calcification into bone tissues.

### Core–shell designs

**Figure 4.**
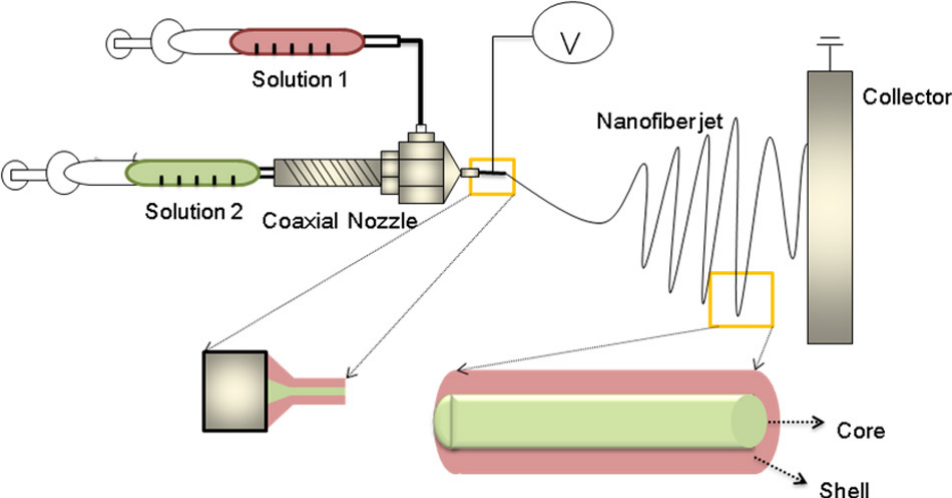
Core–shell nozzle design used to encapsulate drugs within the nanofiber.

One promising nozzle design is the core–shell nozzle, as described in Figure 4. In most cases, the design originates from the need to incorporate drugs inside of the nanofibers. Drugs sheathed inside will be initially protected from environmental factors, such as the solvents used for electrospinning. Furthermore, the encapsulated drugs will be released past the outer shell layer in a more sustainable pattern. This idea has also been proven in some recent studies on bone regeneration, which will be further discussed in section “Development of materials.” Recently, another specific use of the core–shell design for hard tissues has been identified.^16^ A silica-based inorganic phase was overlapped with the inner biopolymer portion to improve the surface properties, such as hydrophilicity and an initial biological response. This system is considered interesting not only for improving cell response but also for its mechanical aspects, as the layered structure of a hard/soft material will generally sustain greater damage, as this has been found in engineering and medical designs like dental crowns.^17^ In fact, the core–shell design produced by electrospinning has great potential for enabling the use of nanofibers in drug delivery systems. Many attempts have been made to encapsulate therapeutics within the core portion of a nanofiber. However, careful designs are required to avoid degradation of the biological molecules to be delivered during the process of electrospinning because such processes require the use of organic solvents (mostly polymers) or treatment with high temperatures (in the case of inorganics). Therefore, the use of water-soluble materials as the core part of the nanofiber is preferred to load drugs safely and to maintain long-lasting biological stability.^18^ The drug release pattern can be influenced by several factors, including the composition and chemical properties of core and shell materials and the shell thickness and degradability. The most commonly used core materials are water-soluble polymers or proteins, including poly(ethylene glycol) (PEG), collagen, and bovine serum albumin (BSA).^19,20^ Poly (ε-caprolactone) (PCL) nanofibers with a core portion consisting of a PEG-incorporating platelet-derived growth factor-BB (PDGF-BB) have shown sustained release for up to 2 months while retaining biological activity of the growth factor, and the release was largely dependent on the molecular weight of the PEG.^19^ When BSA was used as the core material incorporating nerve growth factor ensheathed with a synthetic copolymer of poly(lactic acid) (PLA) and PCL, the growth factor was also well protected and shown to have a near zero-order release pattern over time. This was in direct contrast to the initial burst release over a day observed in the nanofibers made by incorporating growth factor directly within the mixture of BSA/polymer,^20^ thus, confirming that the shell plays a crucial role in establishing the molecular diffusion path and sustaining the release of growth factor.

### Designs for macropore generation

One of the significant challenges in the electrospinning process is the difficulty in creating macropores that are large enough to allow cellular migration and tissue perfusion.^4^ This is particularly an issue in tissue engineering of large-sized defects. The pores to be generated by the electrospinning are well within the range of fiber diameters, that is, tens of nanometers or at most a few micrometers. Some reports have shown the penetration of tissue cells in vitro through the nano/microporous channels of electrospun fibrous mats^21^; however, gaining sufficient cellular penetration for tissue-engineered construct has largely been limited. Tissue penetration in vivo has also been reported in thin tissues such as skin and blood vessels, where the scaffold thicknesses were less than a few millimeters.^22,23^ To tissue-engineer bone tissues with large defect sizes distribution of cells through the depth of nanofibers needs to be improved. Some studies have exploited the generation of macropores within the nanofibrous structure.^24–27^ Nam et al.^24^ attempted to use sodium chloride particles as a porogen. The salt particles were added during the process of electrospinning to be dispersed in the PCL polymer nanofibrous mesh, after which the particles were dissolved to leave porous spaces. After culture for 3 weeks, cell infiltration of up to 4 mm was observed. Another report described using microfibers as a framework of the porous scaffold, on which the nanofibrous mesh was electrospun to form a nanofiber-networked microfibrous scaffold.^25^ Osteoblast-like cell lines and bone marrow stromal cells were shown to favor the nanofiber/microfiber scaffold for their proliferation and production of alkaline phosphatase. Another dual scale scaffold made of nanofibers and microfibers was also designed by combining the processes of polymer melt deposition with electrospinning.^26^ A nanofibrous layer was electrospun upon each layer of the microfiber-structured surface, and the process was repeated to produce a nano–microfibrous scaffold.^26^ While the approach will produce nanofibrous morphology on the microporous scaffold structure, the methodology is considered to have limitations as it is not easily accessible and requires painstaking work to produce large-scale scaffolds. Another report used predesigned electrically conducting molds where the electrospun nanofibers were collected to form a three-dimensional (3D) scaffold network; however, this approach also has limitations for generating thick structures of the scaffolds.^27^ In this sense, the use of thick nanofibrous matrices with 3D complex shapes is still considered a great challenge in realizing hard-tissue engineering using nanofibrous scaffolds.

## Development of materials

### Hybrids and nanocomposites

The materials used in the development of electrospun nanofibers are considered to have properties that are specifically suitable for the calcified hard tissues, in terms of mechanical and biological aspects. The preferred composition would mimic the native ECM, so cells could recognize and utilize the artificial substrate during the regeneration process.^28^ The ECMs of hard tissues typically form a nanoscale organized composite between the inorganic and organic ingredients. While hydroxyapatite (HA) nanocrystals are the major constituent of the composite inorganic materials, the organic constituents are much more variable and depend on the tissues (bone, dentin, and enamel), although fibrous collagen protein forms the main structural network.^29^ Mechanically, the organic network provides resilience, while the inorganic crystals harden the matrix, consequently, contributing to a strong and tough ECM.^30^ The calcification process and the calcified structure are thus the essential traits of hard tissues. Therefore, it is beneficial that the presence of an artificial matrix facilitates the calcification process. The use of bioactive ceramics such as calcium phosphate crystals and glasses with bioactive or soluble compositions has thus assisted in the in vitro and in vivo calcification processes by being directly involved in the formation of the hard tissue.

In this sense, the use of bioactive inorganics with biopolymers (natural or synthetic) is considered a promising strategy to develop artificial matrices for hard-tissue regeneration. Electrospinning of the composite solutions, however, is not easily implemented in the formation of a nanofibrous structure. In many cases, fibers become discontinuous and beads form and destroy continuous nanofibrous morphology, mainly due to the involvement of inorganic crystals. Therefore, one of the biggest considerations in the composite electrospinning process is how to prepare fine nanocrystalline particles and then disperse them homogeneously within the polymer solution. For example, when HA crystals of approximately hundreds of nanometers were directly dispersed in a hydrophobic PLA solution, many large-sized beads were formed, thus limiting the formation of a nanofibrous structure.^31^ However, when HA crystals of tens of nanometers in size were developed and then homogeneously dispersed by using a surfactant that mediates the interface of the hydrophilic nanocrystals and the hydrophobic solution, a uniform-sized, bead-free fibrous morphology could be achieved.^31^ Instead of using HA crystals, ultrafine-sized CaCO_3_ particles have also been successfully incorporated within the biopolymer composition to form an electrospun fiber.^32^ Subsequently, many articles have reported nanocomposite electrospinning using biodegradable synthetic polymers with bioactive inorganic nanoparticulates, such as tricalcium phosphate and bioactive glasses, and most of the nanocomposite nanofibers demonstrated some improvement in the mechanical properties and/or bone cell functions.

The use of natural polymers with inorganics is considered to better mimic the native hard-tissue structure.^33–35^ For this reason, HA was precipitated in situ from the Ca and P precursors within the gelatin or collagen solution, which was then subsequently electrospun into a nanofibrous mesh.^33,35^ High-resolution electron microscopy revealed the ultrafine nanocrystals of a bone mineral–like phase evenly distributed within the natural polymer matrix in a nanofibrous scaffold. The nanofibers containing apatite nanocrystals demonstrated better osteoblastic cell behavior, particularly at the stages of functional differentiation and mineralization.

Although the bioactive inorganics in particle forms were first considered as a composite source of hard-tissue matrices, the size of the particles, their homogeneity/dispersibility, and the added amount have still been an issue to improve for the successful electrospinning. For these reasons, the chemical hybridization of inorganic–organic compositions has also been pursued. As one example, a silicon-based inorganic precursor (glycidoxypropyl trimethoxysilane) was homogenized with a natural polymer gelatin, which was then aged to form siloxane groups and linkages with the amino acids of gelatin to generate a hybridized structure.^36^ The siloxane–gelatin material was electrospun into a continuous nanofiber under adjusted conditions, and the hybrid nanofiber showed an excellent ability to form bone mineral and demonstrated improved osteoblastic activity in vitro, thus proving to be a candidate substrate for bone regeneration.^36^ Another recent study also used synthetic polymer PCL in concert with bioactive glass in a ternary phase, where the PCL solution was mixed with a sol–gel solution based on tetraethyl orthosilicate and then electrospun into a nanofiber.^37^ Although no biological performance was reported in the study, the nanofiber demonstrated hybridization, with hydrogen bonding between the organic and inorganic phases and uniform element distribution.

In the electrospinning process of the hybrid compositions, a certain level of sol–gel reactions, such as the hydrolysis and condensation, is involved. Therefore, careful consideration must be given to controlling the sol properties, which are dependent on time duration. Moreover, care must be given to maintaining the initially generated fibrous morphology of electrospun products without product disintegration, which can be associated with hydration and gelation. Compared to the composite approach, there have been relatively few reports on hybrid nanofibers, which remain of significant interest, because the hybridization process involves chemical reactions at the molecular scale, and thus, the organic–inorganic hybrids often show unexpected performance regarding their physicochemical and mechanical properties.

### Surface mineralization of nanofibers

Many electrospinning materials are chosen from the polymeric compositions rather than from the inorganics, mainly due to mechanical factors. While inorganic nanofibers are generally brittle^38,39^ polymeric nanofibers are flexible and can be easily handled and manipulated for studies. Degradable synthetic polymers are thus preferred for use as a nanofibrous mesh for culturing cells in a variety of areas, including hard tissues. However, the surfaces of synthetic polymers are largely hydrophobic, not providing conditions favorable for cell adhesion.

**Figure 5.**
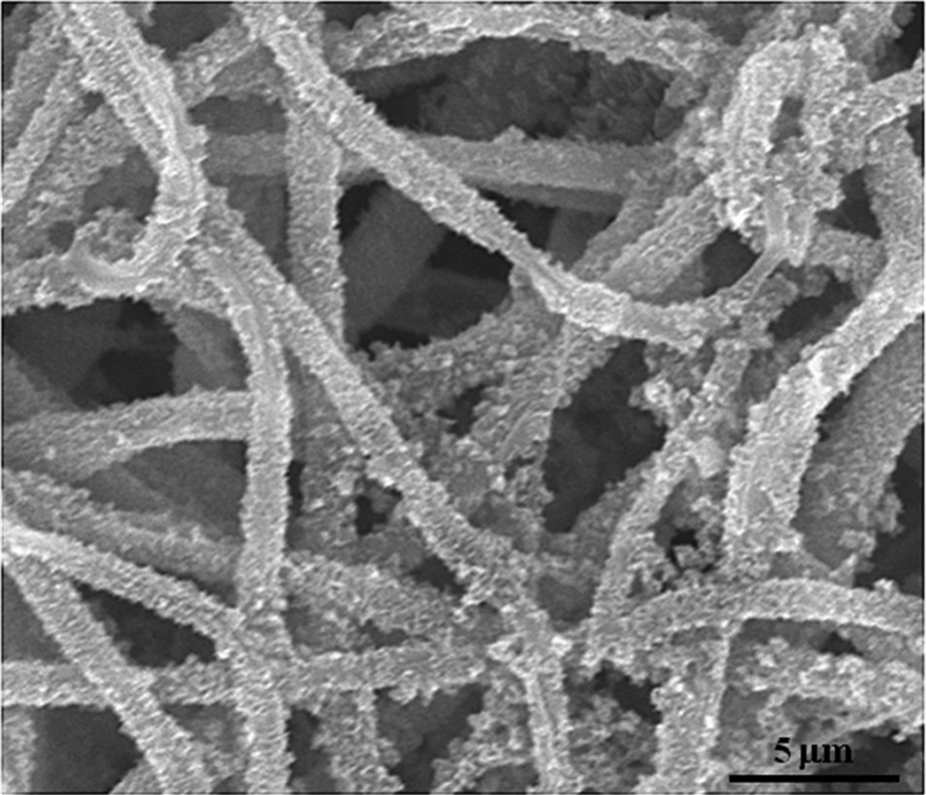
Surface-mineralized PCL nanofiber as a matrix for hard-tissue regeneration. PCL: poly-ε-caprolactone.

One fascinating approach is to modify the surface of the polymer with a bone mineral–like phase, usually calcium-deficient HA.^40–44^ The existence of the mineral phase on the surface of a nanofiber is considered to provide a substrate favorable for bone-related cells, not only in the initial adhesion but also in further matrix synthesis. Compared to natural polymers such as collagen and chitosan, which have numerous ionic molecular groups, the synthetic polymers have relatively few of these groups, which makes it highly difficult to induce mineralization. Thus, the surface treatment of the synthetic polymer nanofibers has been pursued in many different ways. The key is to expose hydroxyl or carboxyl groups by breaking down the polymer chains, and this is possible by treatment in alkaline solution or by irradiation with high-energy sources. The exposed chemical groups are then ready to induce mineralization by further treatment in solutions containing calcium and phosphate ions. One example of a mineralization strategy in PCL nanofibers is shown in Figure 5, where the PCL surface is activated in alkaline solution, followed by calcium phosphate nucleation through soaking in a solution containing calcium and phosphate, followed by further incubation in a body-simulating medium to form a crystallized bone mineral–like phase.^40^ The addition of a natural polymer such as collagen within the synthetic polymer PLA nanofiber was shown to accelerate apatite mineralization on the surface when the fiber was alternatively soaked in calcium and phosphate solutions.^44^ Cui et al.^45^ treated the polymer surface with specifically engineered functional groups, such as hydroxyl, carboxyl, and amino groups, while varying the ratio of each group, and the subsequent mineralization behavior was observed. Depending on the functionalized groups, the crystal size and intensity of the mineralized phase were different, reflecting the importance of the charge density of groups on the fiber surface and the interaction intensities among corresponding ionic groups and/or polar groups. All the mineralized nanofibers were demonstrated to improve the proliferation and osteogenic differentiation of MC3T3-E1 cells, again proving the mineralized nanofibers to be a promising substrate for hard-tissue regeneration.

The existence of bioactive inorganics, such as bioactive glass nanocomponents and calcium phosphate particles within the synthetic polymer nanofibers, has been shown to improve the surface mineralization process.^46^ The inorganic phases can provide nuclei sites for the initiation of mineralization, and the release of ions (such as calcium, phosphate, and silicon) form the inorganics helps the precipitation of surface minerals.

The mineralized surface provides an excellent substrate for the initial adherence and spreading of precursors and/or stem cells and their later growth and differentiation to form bone-specific cells because the surface was functionalized to largely mimic the native bone mineral. Upon the mineralized surface, adhesive proteins are also favored to anchor, thus providing conditions for cells to recognize the surface.^47,48^ Cells supported on the mineralized surface are managed to synthesize ECMs that are more relevant to the development of bone tissue, such as collagen type I, alkaline phosphatase, osteopontin, osteocalcin, and so on. The mineralized surface also plays a direct role in the cellular mineralization process, by providing a mineral source for calcification.

## Applications with therapeutic potential

**Figure 6.**
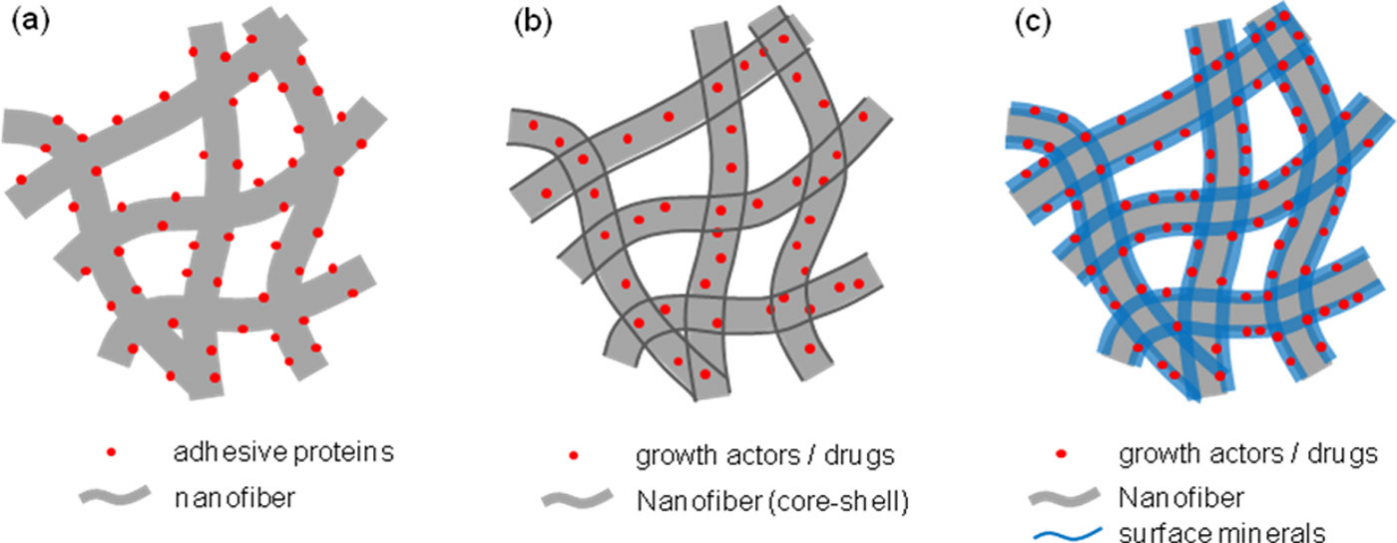
Strategies for utilizing bioactive molecules in concert with nanofibrous matrices for bone regeneration; (a) adhesive proteins tethered on the nanofiber surface, (b) growth factors or drugs incorporated within the nanofiber, and (c) bioactive molecules incorporated within the mineralized surface of the nanofiber.

The potential of biomedical materials for use in hard-tissue regeneration can be greatly improved by combining bioactive molecules, such as native proteins and chemical drugs. The appropriate use of such bioactive molecules is thus one of the key strategies to produce artificial scaffolds with therapeutic efficacy and a tissue-mimicking structure. It is considered that such molecules can be administered on the surface or inside of the nanofibrous matrices. When supported on the surface of the nanofiber, the bioactive molecules will be in contact with the cells and thus be able to regulate initial cellular events. On the other hand, when incorporated within the nanofibers, the molecules inside are protected from the initial biological reactions, and the nanofibers are able to sustainably release a therapeutic substance over long periods of time. In this case, however, special precautions must be taken during the processing of nanofibers to protect the incorporated bioactive substances from being degraded. Specifically for hard tissues, surface-mineralized nanofibers are considered to provide effective substrate conditions for retention of certain therapeutics and subsequent sustained release. Three key strategies to utilizing bioactive molecules are schematically shown in Figure 6.

**Figure 7.**
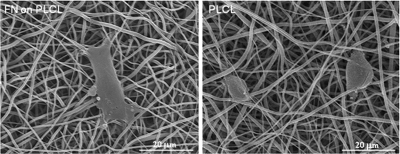
Improvement of cell adhesion on the PLCL nanofiber by surface functionalization with adhesive ligand FN_9–10_ (bone marrow–derived mesenchymal stem cells on FN–PLCL vs. PLCL after 3 h of culture). Cells were more flattened and elongated in spreading behavior on the FN-tethered PLCL nanofiber than those on the pure PLCL nanofiber. PLCL: poly(lactide-*co*-caprolactone); FN: fibronectin.

A great deal of efforts have been given to utilize the adhesive proteins, such as fibronectin (FN), vitronectin, laminin, and collagen, in combination with the biopolymer nanofibers.^49-54^ Those proteins are the key molecules that mediate initial adhesion events in anchorage-dependent cells, which then further regulate intracellular signaling processes that are involved in cell spreading, migration, mitosis, differentiation, and death.^50^ Therefore, the presence of adhesive proteins on the biopolymer nanofibers facilitates initial cell adhesion because most synthetic polymers are highly hydrophobic and lack an adhesive motif, which favors initial cellular events.^51^ However, the direct adsorption of proteins on the synthetic polymer is greatly limited; thus, the surface of the polymer needs to be activated to allow formation of chemical bonds with the proteins. Kim et al.^52^ aminated the surface of a poly(lactide-*co*-glycolide) (PLGA) polymer nanofiber and then coupled it with Gly-Arg-Gly-Asp-Tyr (GRGDY) peptide. They showed a great enhancement of cell attachment, spreading, and proliferation on the peptide grafted nanofiber. The treatment of poly(lactide-*co*-caprolactone) (PLCL) nanofibers with NaOH was also shown to activate the biopolymer surface with numerous carboxyl and hydroxyl groups, which can form covalent linkages with the recombinant FN domain (FN_9–10_) through a carbodiimide cross-linking process.^54^ The initial cellular events, including attachment and spreading, were significantly improved by the treatment, as shown in one of the results in Figure 7.

Apart from the adhesion proteins that are mainly involved in the initial cellular processes, many other bioactive molecules have been implicated to have great impacts on the regeneration processes of bone defects. The most studied and promising of these molecules are the growth factors, which include bone morphogenetic proteins (BMPs), fibroblast growth factors (FGFs), transforming growth factors (TGFs), PDGFs, and insulin-like growth factors (IGFs). Because these proteins are susceptible to denaturation under processing conditions involving high temperatures, low pH, and organic solvents, special attention must be given to the incorporation process within the nanofibers. Therefore, natural polymers that are soluble in water-based solutions are usually favored for delivery of growth factors. Kaplan’s group has explored the delivery of BMP-2 within a silk fibroin–based nanofiber scaffold for bone regeneration.^55^ They directly added BMP-2 to a silk/polyethylene oxide solution either containing HA or not containing HA and then electrospun the solution into fibers. The addition of BMP-2 significantly improved the osteogenic response of human MSCs, as deduced from gene levels and cellular calcification, thus showing the efficacy of BMP-2 within the nanofiber matrix. Although the release behavior of BMP-2 from the nanofiber matrix and the mechanism of action were not detailed in the study, the silk-fibroin nanofibers were proposed as efficient carriers of BMP-2. Casper et al.^56^ used heparin to produce a high affinity for growth factors, including FGF. PEG nanofibers retaining heparin demonstrated a fairly good sustained release of basic FGF (bFGF) during a period of approximately 14 days.

Apart from proteins, nucleic acids may also be delivered by polymer nanofibers. Luu et al.^57^ incorporated plasmid DNA contained within PLA–PEG copolymer nanofibers and showed that the system released 80% of the gene content after 20 days. When the gene was transfected into the osteoblastic cells, an improvement in the transfection efficiency was noticed for the DNA incorporated by using nanofibers compared to the efficiency using a naked plasmid DNA, which was, however, lower than the efficiency obtained using a commercial transfection reagent. Generally, genes are very sensitive to their surrounding biological conditions; therefore, it is important to maintain a stable biological environment. Liang et al.^58^ incorporated DNA into a PLA–PEG–PLA block copolymer during the electrospinning process and showed significantly enhanced transfection efficiency when the cells were cultured on the nanofiber. In a recent similar study, Nie et al.^59^ developed BMP-2 plasmid DNA–chitosan nanoparticles within a PLGA–HA nanofiber matrix for bone regeneration.

In addition to biological molecules, some chemical drugs such as antimicrobial and anticancer agents have been introduced into nanofiber scaffolds to enhance their therapeutic efficacy. Tetracycline hydrochloride for periodontal use was loaded within a mixture of polymers (PLA and poly(ethylene-co-vinyl acetate) (PEVA). The incorporated tetracycline was rapidly released within 10–12 h, and the release rate was controlled by a change in the composition.^60^ The incorporation of hydrophobic drugs such as paclitaxel was made possible by mixing the drug with hydrophobic polymers.^61,62^ In addition to the type of polymer used to encapsulate the drugs, the strength of the polymer–drug interaction also greatly affects the drug release rate.^63^ As an antibacterial agent, silver nanoparticles have been incorporated within polymeric nanofibers.^64^ The silver nanoparticles embedded within the nanofiber matrix exhibited antibacterial efficacy against several types of bacteria.

Compared to the natural polymers, synthetic polymers are usually soluble in organic solvents, which would present harsh conditions for proteins. Therefore, a more general strategy for the safe delivery of proteins without regard to the encapsulating material would employ a core–shell nanofibrous structure composed of two concentric nozzles, as was mentioned in previous section.^65,66^ The core materials are thus water soluble and can safely hold proteins, and the shell portion shielding the encapsulated proteins is favored to be biocompatible to comprise the surface structure of the nanofiber scaffolds. Therefore, design parameters, such as the type of core–shell materials and the thickness and microstructure of the shell, greatly influence the release pattern of the proteins contained inside; likewise, the release behaviors need to be interpreted by those parameters. A recent study described the use of nanofibers for bone regeneration by designing a core–shell structure consisting of a poly(ethylene oxide) (PEO) core incorporating BMP-2, which was encapsulated by a PCL–PEG-blended polymer shell.^67^ Release profiles showed substantial sustained release of BMP-2 and were highly dependent on the blended composition and the pores produced in the shell. Moreover, the core–shell-structured nanofibers incorporating BMPs promoted human MSCs induction into an osteoblastic lineage, as well as in vivo bone regeneration in cranial defects, suggesting the performance of the therapeutically developed nanofibers. Porogen contained in the shell portion of the nanofiber could be used to modulate the drug release kinetics.^68^ The mineralized surface, along with its direct influence on the action of cells related to hard-tissue regeneration, is considered to be a promising focus of research for the capture of biomolecules and drugs, and especially for drugs that have a high affinity for bone minerals. Some examples are proteins such as osteocalcin, which are deeply involved in the in vivo mineralization process. The recognition of HA by osteocalcin is very specific at the molecular level, and thus, the bonds between them are strong.^69^ X-ray crystal structure analysis revealed that a negatively charged protein surface coordinates five calcium ions in a spatial orientation that is similar to that of calcium ions in a HA crystal lattice. Another example can be found in alendronate, which is a well-known drug used for the treatment of osteoporosis. Alendronate has high affinity for the HA crystal by forming bonds between its own phosphate groups and the calcium ions in HA.^70^ Therefore, the administration of these therapeutic biomolecules that have specific affinity to the mineral phase is believed to be another promising methodology, which can be further explored.

Although the idea of using nanofibrous matrices as delivery systems for therapeutic molecules has emerged during the last several years due to new designs of materials and equipment, there have been relatively few studies reported yet, particularly with regard to bone-specific trials and in vivo feasibility. Moreover, realizing therapeutic functions through the delivery systems that are relevant to the in vivo situations is somewhat complicated where many/a series of growth factors and biofactors are involved in the bone repair and regenerative processes. For this reason, the design of nanofibrous matrices for enabling delivery of multiple biofactors is an interesting area to follow.

## Perspectives and conclusions

Significant technological and scientific advances have been made in the field of electrospinning for the repair and regeneration of tissues, including bone. Along with the ability to produce a tissue-mimicking nanofibrous structure, other factors such as ease of setup and versatility in tuning composition and morphology have made it possible to design electrospun nanofibrous matrices for target tissues. The structural and compositional traits of bone ECM, namely, a type of nanocomposite comprised of inorganic and organic phases, account for the specific development of electrospun matrices for bone. Compared to other soft tissues, bone tissue requires cellular mineralization or calcification stages to function in a biologically and mechanically relevant manner.

The technological modifications of apparatus needed for alignment of nanofibers, core–shell design, and micro-/macroporous structuring, which have been successfully developed for other tissue types, are also largely appropriate for creating cellular matrices for bone-tissue regeneration. The alignment status of nanofibrous ECMs should alter their cellular behavior and functions to resemble those of hard tissues, including matrix syntheses and mineral deposition. The use of core–shell nanofibers is highly effective for incorporating bone cell–targeting biomolecules within artificial matrices to better mimic the native tissue structure and to regulate osteogenic cell functions. The production of appropriate tissue-engineered constructs requires further enlargement of nanofibrous pore structures to a level that permits cellular migration and tissue perfusion, which are especially relevant factors when targeting large bone defects. Although some recent studies have attempted to enable 3D shaping and macrochanneling of nanofibrous matrices, the associated scaffolding issues have not been fully solved, especially issues relevant to large-scale production of electrospun matrices.

Along with the technological advances, methods for tailoring of the materials into combined or novel compositions are being pursued to improve the mechanical and biological properties that are appropriate for hard tissues. This may be made possible either by the introduction of inorganic nanocomponents within the polymeric nanofibrous structure or the chemical hybridization of the inorganic–organic components in a one-solution pot. This nanocomposite/hybrid approach is preferred to the single component system because the former has often shown better performance in mechanical and biological aspects, such as retention of stiffness and strength, as well as stimulation of osteogenic differentiation and bone regeneration. However, organization of the composition within a nanofibrous structure requires special care to generate a homogeneous compositional distribution without the disintegration of fiber morphology. Instead of adding inorganic phases within the polymeric matrices, tailoring of the polymer surface with bone mineral–like nanocrystals is a promising approach, which provides a biointerface favorable for biological reactions, including protein adsorption, cellular adherence, and bone differentiation.

Biological performance of the artificial nanofibrous materials can be substantially enhanced when therapeutic biofactors are loaded and appropriately delivered to the site of action. Several designs have been tested for their ability to safely and effectively load the biofactors and deliver them in a sustainable and/or controllable manner. A core–shell-structured nanofiber containing biofactors inside a water-friendly core material is one promising design. Encapsulation of the biofactors within nanocapsules, which are then sheltered by a nanofibrous sheath, is also a possible method that can be used to achieve safe loading and sustainable delivery of the therapeutic molecules. The conjugation of therapeutic drugs through bone-mineral affinity/specificity is another new way to utilize the therapeutic agents in concert with nanofibrous matrices for bone regeneration. The concepts and ideas emanating from this therapeutic approach are still at early stages. Numerous proof-of-concept studies that target specific bone-regeneration themes are still required.
